# Alcohol use disorders in multidrug resistant tuberculosis (MDR-TB) patients and their non-tuberculosis family contacts in Nigeria

**DOI:** 10.11604/pamj.2020.36.321.17118

**Published:** 2020-08-21

**Authors:** Victor Olufolahan Lasebikan, Olusoji Mayowa Ige

**Affiliations:** 1 Department of Psychiatry, College of Medicine, University of Ibadan, Ibadan, Nigeria,; 2 Department of Medicine, College of Medicine, University of Ibadan, Ibadan, Nigeria

**Keywords:** Tuberculosis, alcohol use disoder, comorbidities

## Abstract

**Introduction:**

the main aim of this study was to determine the prevalence and associated factors of alcohol use disorder (AUD) in patients with Multi-Drug Treatment-Resistant Tuberculosis (MDR-TB) compared with their non-tuberculosis control, and its association with disease pattern and associated medical comorbidities.

**Methods:**

MDR-TB patients (128) and their respective caregivers were interviewed in a treatment unit in Nigeria. Diagnosis of AUD was made using the Structured Clinical Interview for DSM-IV Axis I Disorder, information was obtained on the severity of the TB and associated health problems.

**Results:**

prevalence of AUD was (21.9%) and was significantly higher among cases than in controls (2.3%), p = 0.006. Severe TB, OR = 3.33 (1.56-6.83), hematological diseases, OR = 2.34 (1.06-4.33) and HIV/AIDS, OR = 3.01 (1.67-7.01) were the strongest predictors of AUD at 95% CI. **Conclusion:** AUD was highly prevalent in MDR-TB and was associated with certain medical comorbidities and increased severity of the illness.

## Introduction

Tuberculosis (TB) is a leading cause of mortality all over the world, and the Global Burden of Disease Study reported that in 2004 TB was responsible for 2.5% of global mortality of which about 95% of the impact was from developing countries [1].

The relationship between alcohol consumption and TB has a long history and TB had for over three centuries had been recognized as an infectious sequel of chronic alcohol consumption [2]. Although controversial, there is extant literature on the nature of the association between TB and alcohol use disorders [3, 4]. There is a clear and strong link between alcohol use disorder (AUD) and the risk of developing active TB and risk of its recent transmission and a possible pathway is through increased risk of progression from infection to disease [3], however, a temporal association is conceivable since alcohol use disorder takes several years to develop, preceding the onset of TB [5]. Furthermore, a large case-control study among the Russian population found a dose-response relationship between alcohol consumption and TB [6]. Another pathway is via increased susceptibility to TB in patients with alcohol use disorders because of immunosuppression [7], yet social marginalization and low socioeconomic status are common to alcohol use disorder [8] and TB [3]. Immunosuppression, poor compliance with anti-TB regime or inadequate treatment leads to the emergence of drug resistant TB [9]. There is a wide range in the prevalence of alcohol use disorder among TB patients with figures ranging 10% through 50%, in Australia, Canada, Russia, Switzerland, and the United States [3]. In low and middle-income countries, rates also vary greatly, 14.9% to 32% in India [10-12], 14-24% in Brazil [13, 14] and 31-62% in South Africa [15].

In Nigeria, south of Sahara, given reports from the World Health Organization ranking the country as the second highest among the countries with TB burden in Africa, there is a lack of available data on AUDs in TB. This may be because physicians are less likely to routinely screen for alcohol use among such population, talk less of implementation of any intervention. Thus, our main aim was to determine the prevalence and associated factors of alcohol use disorders in patients with MDR-TB, compared with their non-tuberculosis contacts. We also determined the predictors of alcohol use disorders in TB. We hypothesized that patients with tuberculosis would have a higher prevalence of AUDs than the control group.

## Methods

### Study design

In this ongoing non-matched case-control study, we recruited the study samples between January 2010 and January 2016.

### Place of study

The study took place at the MDR-TB treatment centre, department of medicine, University College Hospital, Ibadan. This is a 25 bedded special unit, established in Nigeria as the first MDR-TB treatment centre in the country which was established in January 2010. According to the treatment guideline, patients with positive culture and drug susceptibility test (DST) results for multidrug-resistant (MDR)-TB patients were on admission for the first 6 months intensive phase treatment period, followed by a 12-month ambulatory phase. The standard regime during the intensive phase comprises of Amikacin, Levofloxacin, Pyrazinamide, Prothionamide, Cycloserine and Pyridoxine. This was in accordance with the National Tuberculosis Control Program (NTCP), during the ambulatory period, except for Amikacin, all these drugs were continued.

### Ethical consideration

In accordance with the ethical principles of the Committee on Publication Ethics (COPE), ethical approval was obtained from the ethical review committee of the Research Division of the University College Hospital, Ibadan. We also obtained written informed consent from all participants, including the caregivers. Confidentiality was also maintained during the course of the study.

### Study sample

This is an ongoing study that commenced in 2010. We interviewed 128 patients attending the MDR-TB treatment centre and 128 family caregivers who served as non-matched controls. All the patients who had received treatment at the MDR-TB treatment centre since it was established in January 2010 up to January 2016, were recruited into the study. Also recruited were their respective caregivers; all cases and control were consecutively recruited.

### Inclusion

The cases were the patients with tuberculosis attending MDR-TB (pulmonary), while the caregivers served as controls. In this study, a caregiver was defined as a person who was involved with the everyday care of the case and would be very likely to respond to any request for special assistance at any time, if such a request was made by the case. He could be a family member or not [16].

### Exclusion

Participants who were not literate in the languages of instruction during the study (English and Yoruba languages), were excluded. Also excluded were caregivers with a past or current history of tuberculosis.

### Measures

#### Socio-demographic questionnaire

We obtained information on age, gender, marital status, education, occupation, ethnicity, duration of tuberculosis, and severity of TB.

### Structured clinical interview for DSM-IV Axis I Disorder (SCID)

The alcohol section of the substance use disorder module of the Structured Clinical Interview for DSM-IV Axis I Disorder (SCID) [17], was used to obtain a past-month diagnosis of alcohol abuse or dependence. Past- month alcohol use in the current study referred to use in the 4 weeks preceding admission into the MDR-TB unit The SCID is useful by the clinician as part of a normal assessment procedure to confirm a particular diagnosis and the instrument covers all the criteria of the diagnosis as included in the various modules. It is available in a patient edition for use with subjects who have been identified as psychiatric patients and in a non-patients edition which is suitable for use in epidemiological studies.

### Multi Drug Resistant Tuberculosis (MDR-TB)

In the MDR-TB treatment unit, the primary MDR-TB and secondary MDR-TB cases are attended to. Patients who had never been exposed to the anti-TB medications, but acquired the disease by contacts with MDR-TB cases are referred to as the primary MDR-TB cases, while treatment failure cases, relapses, and defaulters and those who require re- treatment are referred to as secondary MDR-TB cases and second line anti-tuberculosis medications are their drugs of choice (World Health, 2010).

### Severity of TB

The National Tuberculosis Program and WHO guidelines which are based on the extent of disease, bacillary load and anatomical site that carries a significant acute threat to life or a risk of subsequent severe handicap, or both are used to classify cases based on severity (National Tuberculosis Programme and WHO guidelines).

### Extent of TB

Extent of the TB was classified as follows: mild (located to a zone), moderate: located to more than a zone, but one side of the lung and severe was classified as both lungs affected (National Tuberculosis Programme and WHO guidelines).

### Category of TB

According to the National Tuberculosis Programme and WHO guidelines, Category 1 TB was defined as freshly diagnosed smear-positive pulmonary TB, smear negative pulmonary TB with extensive parenchymal involvement or new cases with severe forms of TB, e.g. military TB, tuberculous meningitis, tuberculous pericarditis, tuberculous peritonitis, intestinal TB, genitourinary TB, bilateral or extensive TB pleurisy, spinal disease with neurological complications. Category II TB was defined as relapsed and treatment failure (smear-positive) cases; and treatment after substantial interruption [18].

### Medical comorbidity

A checklist of common medical comorbidities associated with MDR-TB was included in the current study. The checklist is based on routine physical examination and laboratory findings by medical specialists managing such physical illnesses and documented in the patients´ medical records. These include hematological diseases, heart failure, pleural effusion, hypertension, diabetes, cancer, glaucoma, deafness, lung abscess, renal failure, arthralgia, gastritis, HIV/AIDS, emphysema and bronchiectasis. The checklist was similarly used in our previous study [19].

### Pre-test

A pre-test was carried out using all instruments of data collection in June 2009, at the State Hospital, Ring Road, Ibadan among a group of 15 patients suffering from chronic obstructive airway disease and their respective caregivers. This was to determine the feasibility of the study and the applicability of the research instruments.

### Analyses

We initially recruited 131 patients attending the MDR-TB treatment centre and 131 caregivers (control). However, data were complete for 128 patients and 128 controls among whom final analyses were carried out. The Chi-square test was used to determine significant differences in binary variables between the cases and the control and conditional logistic analyses for multilevel categorical data. Primary outcome (dependent) variable was alcohol use disorder in MDR-TB patients, while sociodemographic factors and clinical factors such as extent of disease, stage of disease and classification were independent variables. Binary logistic regression analysis was carried out to determine the effect multiple confounding independent variables, using variables that were significantly associated with the primary outcome variables during the initial univariate analysis, p < 0.05. Gender was adjusted for because of the known association between AUD and male gender. The level of statistical significance was set at 0.05, 95% confidence interval for all other analyses. All analyses were carried out using SPSS 20.0.

## Results

In this study, 128 patients with MDR-TB were compared with 115 non-tuberculous accompanying family persons/caregivers. The median age of the patients was 34 and 48 years for the caregivers, Z = -5.7, p < 0.001. Compared with the control group, a higher proportion of the MDR-TB cases were men, X^2^ = 14.2, p < 0.001 and a higher proportion of the MDR-TB cases were unmarried , X^2^ = 7.7, p = 0.003 and were also unemployed, X^2^ = 4.6, p = 0.03. Prevalence of past year alcohol use disorder (AUD) was 28 (21.9) among MDR-TB cases and 3 (2.3%) among the control (Table 1). Post-hoc pairwise comparisons show that significantly fewer respondents of the Yoruba ethnic group 7 (8.2%) had AUD compared with the Hausa ethnic group (29.4%), X^2^ = 6.1, p = 0.01, also when compared with the Igbo ethnic group 8 (53.3%), X^2^ = 20.1, p < 0.001, and when compared with the minorities 8 (72.7%), FE p value < 0.001.

**Table 1:** socio-demographic characteristics of patients and controls

Demography	Patients (N = 128)		Control (N = 128)		X^2^	P
	**N**	**%**	**N**	**%**		
**Age**						
<25	20	15.6	16	12.5	21.0 (df,4)	< 0.001
25-34	44	34.4	26	20.3		
35-44	33	25.8	28	21.9		
45-54	26	20.3	31	24.2		
>54	5	3.9	27	21.1		
Median Age	34		48		-5.7Z	< 0.001
**Gender**						
Male	49	38.3	22	17.2	14.2	< 0.001
Female	79	61.7	106	82.8		
**Education**						
No formal	67	52.3	90	70.3	11.0 (df,5)	0.05
Some primary	22	17.2	13	10.1		
Primary	23	18.0	11	8.6		
Some secondary	9	7.0	9	7.0		
Secondary	6	4.7	5	4.0		
Post-secondary	1	0.8	-	-		
**Marital status**						
Married	45	35.2	67	52.3	7.7	0.003
Unmarried	83	64.8	61	47.7		
**Employment**						
Employed	46	35.9	63	49.2	4.6	0.03
Unemployed	82	64.1	65	50.8		
**Religion**						
Christianity	60	46.9	54	42.2	0.5	0.5
Islam	68	53.1	74	57.8		
**Ethnicity**						
Yoruba	85	66.4	84	65.6	0.1 (df,3)	0.99
Hausa	17	13.3	18	14.1		
Igbo	15	11.7	14	10.9		
Minority Tribes	11	8.6	12	9.3		
**Alcohol Abuse**						
Yes	26	20.3	3	2.3	20.6	< 0.001
No	102	90.6	125	97.7		
**Alcohol Dependence**						
Yes	2	1.5	-	-	-	-
No	126	98.5	128	100.0		
**Alcohol Use Disorder**						
Yes	28	21.9	3	2.3	7.6	0.006
No	100	78.1	125	97.7		

Z: Wilcoxon Signed Rank Test

Among the cases, AUD was more prevalent in men, 24 (49.0%), X^2^ = 34.1, p < 0.001, the unmarried, X^2^= 6.9, p = 0.009, the Christians, X^2^ = 13.3, p < 0.001 and less prevalent among the Yoruba Ethnic group, X^2^ = 35.1, p < 0.001 (Table 2). Among patients with MDR-TB, there were significant associations between AUD and comorbid hematological diseases, X^2^ = 11.4, p = 0.001; HIVAIDS, X^2^ = 6.2, p = 0.01; category 2 TB, X^2^ = 5.9, p = 0.02 and extent of TB, X^2^= 20.3, p < 0.001 (Table 3). Predictors of AUD in MDR-TB were being unmarried, OR = 1. 67, 95% CI (1.16-3.04, moderate TB, OR = 1.56, 95% CI (1.11-3.56), severe TB, OR = 3.33, 95% CI (1.56-6.83), hematological diseases, OR = 2.34, 95% CI (1.06-4.33) and HIV/AIDS, OR = 3.01, 95% CI (1.67-7.01) (Table 4).

**Table 2:** demographic correlates of alcohol use disorder (AUD) in tuberculosis

Demography	Cases N = 128		AUD N = 28		X^2^	P
	**N**	**%**	**N**	**%**		
**Age (Years)**						
< 25	20	15.6	8	40.0	9.9 (df,4)	0.04BNS
25-34	44	34.4	7	15.9		
35-44	33	25.8	6	18.2		
44-54	26	20.3	4	15.4		
>54	5	3.9	3	80.0		
**Gender**						
Male	49	38.3	24	49.0	34.1	< 0.001
Female	79	61.7	4	5.1		
**Marital Status**						
Married	45	35.2	4	8.9	6.9	0.009
Unmarried	83	64.8	24	28.9		
Employment						
Employed	46	35.9	9	19.6	0.2	0.6
Unemployed	82	64.1	19	23.2		
Religion						
Christianity	45	35.2	18	40.0	13.3	< 0.001
Islam	83	64.8	10	12.0		
**Education**						
No formal	67	52.3	8	11.9	9.0	0.1
Primary uncompleted	22	17.2	8	36.4		
Primary	23	18.0	7	30.4		
Secondary Uncompleted	9	7.0	3	33.3		
Secondary	6	4.7	2	33.3		
**Post-Secondary**	1	0.8	-	-		
Ethnicity						
Yoruba	85	66.4	7	8.2	35.1	< 0.001BS
Hausa	17	13.3	5	29.4		
Igbo	15	11.7	8	53.3		
Minorities	11	8.6	8	72.7		

BNS: Not Significant after Bonferonni Adjustemsnt: BS: Significant after Bonferonni Adjustment:

**Table 3:** clinical correlates of AUD in tuberculosis N = 128

Clinical variables	Present	Cases	N= 128	AUD	N = 28	X^2^	P
		**N**	**%**	**N**	**%**		
Hematological diseases	No	114	88.7	20	17.4	11.4	0.001
	Yes	14	11.3	8	57.1		
Heart Failure	No	122	95.7	25	20.5	0.6	0.5
	Yes	6	14.3	2	33.3		
Pleural Effusion	No	91	71.3	21	23.1	0.3	0.7
	Yes	37	18.7	7	18.9		
Hypertension	No	110	85.9	23	20.9	0.4	0.5
	Yes	18	14.1	5	27.7		
Diabetes	No	109	85.5	26	23.9	0.07	0.8
	Yes	19	14.5	4	21.1		
Cancer	No	125	98.3	27	21.6	0.2	0.6
	Yes	3	1.7	1	33.3		
Glaucoma	No	116	90.6	25	21.6	0.2	0.7
	Yes	12	9.4	3	25.0		
Deafness	No	89	69.6	20	22.5	0.06	0.8
	Yes	39	10.4	8	20.5		
Lung Abscess	No	108	84.3	24	22.2	0.08	0.7
	Yes	20	15.7	4	20.0		
Liver disease (hepatitis)	No	86	67.2	13	15.1	3.9	< 0.05
	Yes	42	32.8	14	33.3		
Renal Failure	No	112	87.5	25	24.5	0.1	0.7
	Yes	16	12.5	3	18,8		
Arthralgia	No	89	69.6	21	23.6	0.5	0.5
	Yes	39	10.4	7	17.9		
Gastritis	No	67	53.0	18	26.9	2.1	0.2
	Yes	61	47.0	10	16.4		
HIV/AIDS	No	110	85.2	20	18.8	6.2	0.01
	Yes	18	14.8	8	44.4		
Empyema	No	119	93.0	26	21.8	0.06	1.0
	Yes	9	7.0	2	22.2		
Bronchiectasis	No	117	91.3	26	22.2	0.09	0.8
	Yes	11	8.7	2	18.2		
Extra pulmonary	No	83	65.8	20	24.1	0.6	0.4
	Yes	45	34.2	8	17.7		
Category	I	84	66.1	13	15.5	5.9	0.02
	II	44	33.9	15	34.1		
Extent/Severity of TB	Mild	42	32.8	4	9.5	20.3 (df,2)	< 0.001
	Moderate	60	46.9	10	16.7		
	Severe	26	20.3	14	53.8		

Haematological diseases :anaemia; FE: Fishers Exact P value; BS: Significant after Bonferonni adjustment; Mild: located to a zone, moderate: more than a zone but one side of the lung, severe: both lungs; Category 1: freshly diagnosed smear-positive pulmonary TB, smear negative pulmonary TB with extensive parenchymal involvement; or new cases with severe forms of TB, e.g. military TB, tuberculous meningitis, tuberculous pericarditis, tuberculous peritonitis, intestinal TB, genitourinary TB, bilateral or extensive TB pleurisy, spinal disease with neurological complications; Category II TB: relapsed and treatment failure (smear-positive) cases; and treatment after substantial interruption. These patients are at risk of developing multidrug resistant TB (MDR-TB)

**Table 4:** predictors of past month AUD in tuberculosis

Independent Variables	Exp (B)	95% CI		P
		**Lower Bound**	**Upper Bound**	
**Marital Status**				
Married	1			
Unmarried	1.67	1.16	3.04	0.01
**Religion**				
Christianity	1			
Islam	1.81	0.09	4.06	0.06
**Ethnicity**				
Yoruba	1			
Hausa	1.56	0.87	3.07	0.09
Igbo	1.99	0.82	4.11	0.08
Minorities	2.12	0.97	4.99	0.06
**Category**				
I	1			
II	1.95	0.78	3.81	0.09
**Extent/Severity of TB**				
Mild	1			
Moderate	1.56	1.11	3.56	0.04
Severe	3.33	1.56	6.83	0.001
**Haematological Diseases**				
No	1			
Yes	2.34	1.06	4.33	0.03
**HIVAIDS**				
No	1			
Yes	3.01	1.67	7.03	0.003

Extent: Mild: located to a zone, moderate: more than a zone but one side of the lung, severe: both lungs; Category 1: freshly diagnosed smear-positive pulmonary TB, smear negative pulmonary TB with extensive parenchymal involvement; or new cases with severe forms of TB, e.g. military TB, tuberculous meningitis, tuberculous pericarditis, tuberculous peritonitis, intestinal TB, genitourinary TB, bilateral or extensive TB pleurisy, spinal disease with neurological complications; Category II TB: relapsed and treatment failure (smear-positive) cases; and treatment after substantial interruption. These patients are at risk of developing multidrug resistant TB (MDR-TB)

## Discussion

In this study, which aimed to determine the prevalence of AUD in MDR-TB, we found that 21.9% of patients with TB and 2.3% of caregivers had AUD. We also found that medical comorbidities, being unmarried and the extent of the TB were predictive of AUD in MDR-TB. This is the first report on this subject in Nigeria, sub-Saharan and the discussion of this report is presented herein. The prevalence of 21.4% of AUD reported in the current study is lower than the figure reported in Zambia, East Africa (34.7%) [20], the figure (36%) reported in South Africa [15], and the 55.1% reported in Russia [21], but higher than the figure reported in India (14.9%) [12]. There are several plausible reasons for the high rate of AUD in our MDR-TB sample, firstly, is that patients with MDR-TB are more likely to have treatment resistance because of persistent or continued drinking, secondly is that AUD influences the outcome of uncomplicated TB one of which is the development of treatment resistance [5], specifically, there are strong evidence of a negative impact of AUD on the clinical course of TB, [22] including progression to the most destructive form [23]. It is conceivable that the co-occurrence of AUD with MDR-TB in Nigeria is a huge public health issue, given the negative impact of alcohol on the immune system, leading to either susceptibility to other diseases, re-infection, treatment default or to altered pharmacokinetics of medicines used in the treatment of the TB [4]. Furthermore, excessive drinking may account for a high TB mortality as it may lead to TB treatment failure and medical comorbidities [4, 24, 25]. We found that AUD was more prevalent in men and the unmarried. These are in support of the results of O´Connell *et al*. in Zambia [20] and Suhadev *et al*. in India [12]. This observation is not peculiar to the current study population, but are common demographic correlates of excessive drinking across in different population [26, 27]. The finding that AUD was more prevalent among the Christians is in support of previous studies in Nigeria [26, 28]. This may be adduced to the general condemnation of alcohol consumption in Islam [29]. The association of ethnicity with AUD as reported in the current study in which the Yoruba ethnic groups, compared with other ethnic groups were less likely to have AUD was also noted in a previous study among the military population [30]. Factors mediating this ethnic variations are not clearly known, however, there are reports that drinking is more embedded within certain cultures in Nigeria such as the middle belt, south-south of Nigeria and the minority tribes [31].

### AUD and Medical Comorbidities in MDR-TB

Our study sample is generally characterized by high rates of medical comorbidities spanning different disease conditions. Notable is the association between hematological diseases, HIV/AIDS and to a lesser extent liver disease and AUD. The presence of medical comorbidities among the current sample has two interrelated dimensions, one is that AUD is an independent cause of medical comorbidities so also is MDR-TB. The association between TB and HIV/AIDS is very common [32, 33] and is a subject of utmost public health concern because of an acceleration in the deterioration of immunological functions, thereby leading to premature mortality if untreated most especially in developing countries [32] where 99% of the deaths occur [33]. It is conceivable that among patients with AUD, the outcome will be gloomier. Studies suggest that alcohol use may be associated with increased sexual risk-taking behaviours, or that sexual risk or conversely, those with HIV infection turn to alcohol [34]. In support of previous studies [35, 36], we also found an association between AUD and hematological diseases. Such hematological disorders could have arisen secondary to the TB itself through a variety of mechanisms and may affect red cells, white cells, platelets, and the coagulation system [35] or could be a sequel of the AUD. For example, haematological dieases such as haemolytic anaemia [37, 38] or nutritionally induced [39] are very common in AUD. Thus, the occurrence of AUD in MDR-TB is likely to be associated with a higher likelihood of hematological problems. We found that liver diseases were significantly associated with AUD. In addition to the high prevalence of liver diseases in TB [40], patients with AUD have been found to suffer from a number of liver diseases due to liver injury [41-43]. Thus, the occurrence of alcohol related liver disease with MDR-TB creates a double dose jeopardy for the patient.

### AUD and Severity of MDR-TB

We found that AUD was significantly more reported among those with category 2 tuberculosis and also among those with moderate and severe TB. These findings are in support of previous studies [23, 44, 45]. Several factors may be responsible for this association. Firstly, is that alcohol abuse or dependence is a risk factor for frequent interruption or compliance with medications [46], secondly, the risk of developing antituberculous-induced adverse reaction such as hepatotoxicity [47], the probability of which is higher in AUD, thirdly, is the tendency of developing alcohol related medical comorbidities [48] and the potential management difficulties as a result of potential drug interactions from multiple pathologies. This leads to disease progression and high mortality.

### Regression analysis

Regression analysis shows that being unmarried, moderate or severe TB, haematological diseases and HIV/AIDS remained as predictors of AUD after adjusting for gender. Our findings also suggest the need for a comprehensive multidisciplinary treatment approach for patients with MDR-TB in view of the associated medical comorbidities. Important is the provision of alcohol screening and cessation programs while patients are receiving treatment for tuberculosis in primary care. This has the potential likelihood of identifying problem drinkers and initiating a treatment before the tuberculosis progresses into multi-drug resistance. Treatment adherence should be emphasized for patients at the early stage of tuberculosis in order to minimize the progression of the disease. For example, the Directly Observed Therapy Short Course (DOTS), which is also practiced in Nigeria [49] should remain a major way of ensuring treatment adherence. A prevalence of 2.3% of AUD among caregivers of patients with MDR-TB is an important finding. This is very relevant, given reports that AUD within the community sample had a very low prevalence [28]. The high prevalence of AUD among the caregivers may suggest the use of alcohol as a coping mechanism for the caregivers. Thus, these caregivers require follow-up.

### Implications of the current study

Our findings, therefore, underscore the need for screening and brief intervention for patients not only in MDR-TB, but also in non MDR-TB treatment units. For example, Mathew and co-investigators established a management algorithm which enabled patients with AUDIT score 8 and above be referred for evaluation and treatment since it is not likely for TB physicians to have training and time to deliver addictions care [21]. This type of addiction service could be extended to other psychoactive substances and have the potentials of improving treatment outcomes and prognosis of tuberculosis. There is also the need for a larger scale, multi-centre follow-up study, with a sample size capable to yielding more informative results. This study has a number of limitations. Our findings may not be generalizable to other MDR-TB treatment facilities in Nigeria or other non- multidrug resistant tuberculosis treatment centres. This should be considered in interpreting our findings from this study. Our study has not also established a causal relationship between AUD and MDR-TB. Future prospective study designs may be able to be more inferential on the subject.

## Conclusion

The rate of AUD in MDR-TB, according to this report is high. The sample was also characterized by a high rate of medical comorbidities such as haematological problems, HIV/AIDS and liver problems. They also had a high rate of complications such as extrapulmonary spread and moderate or severe illness.

### What is known about this topic

- Excessive alcohol consumption is associated with tuberculosis;
- The prevalence of alcohol use disorder is higher in tuberculosis than in the general population.

### What this study adds

- Moderate or severe tuberculosis were associated with alcohol use disorder;
- Haematological diseases and HIV/AIDS were associated with alcohol use disorder.
